# T-helper 1 immunoreaction influences survival in muscle-invasive bladder cancer: proof of concept

**DOI:** 10.3332/ecancer.2014.486

**Published:** 2014-12-01

**Authors:** Alexandre Ingels, Rafael E. Sanchez Salas, Vincent Ravery, Gaëlle Fromont-Hankard, Pierre Validire, Jean-Jacques Patard, Géraldine Pignot, Dominique Prapotnich, Fabien Olivier, Marc Galiano, Eric Barret, Francois Rozet, Nina Weber, Xavier Cathelineau

**Affiliations:** 1 Institut Montsouris, Department of Urology, 42 Boulevard Jourdan, 75014 Paris, France; 2 Hôpital Bichat, Department of Urology, 46 Rue Henri Huchard, 75018 Paris, France; 3 Hôpital Bretonneau, Department of Pathology, 2 Boulevard Tonnelé, 37000 Tours, France; 4 Institut Montsouris, Department of Pathology, 42 Boulevard Jourdan, 75014 Paris, France; 5 Hôpital Bicêtre, Department of Urology, 78 Rue du Général Leclerc, 94270 le Kremlin-Bicêtre, France; 6 ALTRAN, Department of Statistics, 2 Rue Paul Dautier, 78140 Véllizy-Villacoublay, France

**Keywords:** bladder cancer, immune reaction, immunohistochemistry, markers, prognosis, tumor infiltrating lympocytes, immunoscore

## Abstract

**Objective:**

To define immunoscore in bladder cancer studying T helper 1 (Th1) immunoreaction. To define a cancer-specific survival model based on Th1 cells infiltration.

**Methods:**

A total of 252 patients underwent primary transurethral resection of bladder tumour at our Institution. A retrospective review of a selected cohort with pT1 and muscle-invasive bladder cancer (MIBC) lesions was performed. Pathology blocks were marked with CD3 and CD8 antibodies. Immune cells density in stromal reaction (SR) was measured on five distinct high-power field (HPF) by two dedicated uro-pathologist blinded for patients’ evolution.

**Statistics:**

Student test or non-parametric Wilcoxon test as appropriate to compare means between two groups. Receiver operating characteristics (ROC) curve to define markers threshold. Cox model to assess survival’s predictors.

**Results:**

Ten pT1 and 20 MIBC consecutive cases were analysed. Median follow-up was 33.4 months. Immunohistological analysis for pT1 lesions featured limited SR. For MIBC, the mean density of lymphocytes in the SR was of 105/HPF (CD3) and 86/HPF (CD8). Survivors harboured higher lymphocytes densities versus non survivors (CD3: p = 0.0319; CD8: p = 0.0279). CD3 (p = 0.034) and CD8 (p = 0.034) lymphocytes densities were independently associated with cancer-specific survival on Cox model analyses. The retrospective design and small size of cohorts are the study limitations.

**Conclusions:**

High CD3 and CD8 lymphocytes SR densities are associated with better cancer-specific survival for MIBC. Th1 reaction against the tumour seems to be protective for bladder cancer. Further evaluation is warranted.

## Introduction

Immune responses play an important role in bladder cancer and, in particular, with regard to its treatment. In 1976, Morales *et al* first reported the use of intravesical Bacillus calmette-guérin (BCG) therapy for non–muscle-invasive bladder cancer (NMIBC) [1]. BCG was the first US Food and Drug Administration (FDA)-approved immunotherapy and has been consistently demonstrated to reduce the risk of bladder cancer recurrence by eliciting a strong cellular immune response, initially directed against the attenuated mycobacteria but ultimately targeting bladder tumour cells [2, 3]. The precise immune mechanism leading to the host’s defense against bladder tumour is still not completely understood [4]. However, different reports from the literature have emphasised the key role of T-helper 1 (Th1) cytotoxic cells, including tumour-infiltrating lymphocytes (TILs) T3 and T8 [5].

It is therefore logical to investigate whether the measurement of the Th1 immunoreaction could be used as a potential predictor of recurrence and progression in NMIBC or survival in MIBC. Most predictive factors in bladder cancer are based on clinical features such as tumour node metastasis (TNM) stage and tumour grade. Their limitations have been extensively described [6] and there is wide intraobserver variability from one pathologist to another in tumour grading from transurethral resection of bladder tumour (TURBT) specimens [7].

We hypothesise here that information from the host’s response against the tumour would possibly complement that provided by the tumour characteristics.

Sharma *et al* previously reported on the specific cytotoxic immune response directed against bladder cancer and its clinical prognostic role [8].

The aim of this study was to explore the feasibility of an immune score (‘immunoscore’) focusing on the Th1 cytotoxic immune reaction in pT1 NMIBC and MIBC. We used the specific markers CD3 and CD8 as recommended by a recently published consensus-based recommendation on how to design immunoscore to classify cancer [9].

## Materials and methods

### Population

This was a single-centre retrospective study. From October 2005 to May 2010, 252 patients from the Pathology Department database at the Institut Montsouris underwent a first TURBT with a pT1 or MIBC diagnosis. We selected a cohort from this population to assess the feasibility of an immunoscore. Inclusion criteria were a confirmed urothelial carcinoma, invasion at least up to the lamina propria (≥pT1), and no previous BCG or chemotherapy treatment. Additional information such as clinical data, pathologic reports from TURBT, and follow-up data (second look, chemotherapy or BCG instillation, surgery, radiation therapy, systemic chemotherapy, recurrence, progression, specific and overall mortality, and follow-up time) was recorded in a database. A total of 30 patients (ten consecutive treatment-naive patients with NMIBC and 20 consecutive treatment-naive patients with MIBC) met the inclusion criteria and made up the population of the study.

### Pathology

After pT stage confirmation via a regular histologic reading of hematoxylin and eosin-stained slides by our trained pathologist, new 4-μm thick slides containing the tumour and invasive margins were prepared using a microtome (Finesse, Thermo Fischer Scientific, Waltham, MA, USA). Deparaffinization, rehydration, and immunostaining were performed automatically (Benchmark Ultra, Roche, Basel, Switzerland). For the detection of CD8 cells, murine antihuman clone C8/144B (Dako, Glostrup, Denmark) was used; for the detection of CD3 cells, rabbit antihuman CD3 2GV6 (Ventana, Oro Valley, AZ, USA) was used. Those specific antibodies were chosen after a recent consensus that found reproducibility, little background noise, and applicability for immunoscore development [9]. For each specimen, five independent HPF (10 × 40) areas with the most abundant CD8 or CD3 tumour infiltrates were selected and imaged digitally with a Nikon Coolpix 990 camera with standard commercial software (Nikon, Melville, NY, USA). Because the material was from TURBT, the central tumour was not clearly defined, and we decided to focus on the invasive margin SR of the tumour. Tumour infiltrating lymhpocytes (TILs) were counted manually from the digital images displayed on the monitor. All counts were repeated twice by the same investigator and confirmed by a second investigator, both blinded for patients’ clinical outcomes. The average of the repeat counts was used for statistical analyses.

### Statistics

The statistical analysis was performed using Stata software (StataCorp, College Station, TX, USA). The correlation between two continuous data sets was tested with the Pearson correlation coefficient. Normal repartition of the data was tested with the Shapiro–Wilk test. A comparison of means equity between two continuous data was done with the Student *t* test when normal repartition was confirmed or with the nonparametric Wilcoxon test, otherwise. Log-rank analysis was performed to compare survival probability between two groups. The threshold to transform a continuous data to a positive/negative nominal value for marker measurement was set using the shortest Euclidean distance from the ROC curve to the top left of the graph. The Cox model was used to test a variable as a risk factor for survival.

## Results

### Population

The selected cohort was composed of 30 patients: a total of 10 pT1 and 20 MIBC. Table 1 lists their characteristics. There was no metastatic stage based on pretreatment computed tomography (CT) evaluation. The median age was 68.6 years. All tumours were high grade according to the 2004 World Health Organisation classification. Seventeen patients (57%) had a recurrence. Fifteen patients (50%) progressed from pT1 to MIBC or from MIBC to metastatic stage. Among patients with MIBC, 12 (60%) died during follow-up; cancer was always the cause of mortality. For pT1 tumours, two patients (20%) died, both from bladder cancer. Median follow-up was 33.4 months (range: 6.6–64.4 months).

### Immunohistochemistry

**Figure 1.**
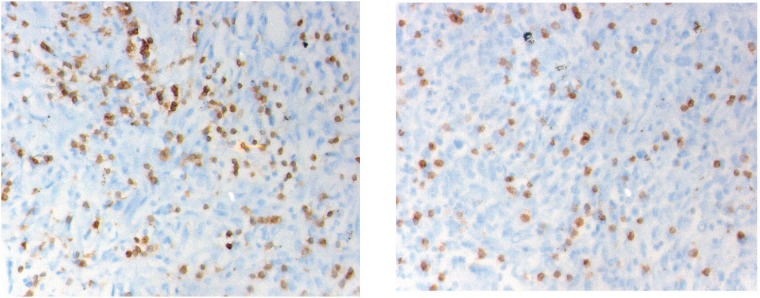
Immunostaining of the CD3 (left) and CD8 (right) cells infiltration into the peri-tumoural stroma (magnification 40 x 10). The cell density is counted in five distinctive fields. The final value is the five figures’ mean.

CD3 and CD8 TILS were detected in both SR and epithelial tumour nests (Figure 1). As stated previously, we decided to focus only on the SR.

For pT1 tumours, the small invasive margin surface made it difficult to obtain reproducible results using a standard manual technique. We did not consider this technique feasible under these conditions. Only the 20 MIBC patients were included in the analysis.

In MIBC, assessment of the infiltration of CD3 cells showed a mean density of 104 cells/HPF; median, 83 cells/HPF; range 3–312 cells/HPF; standard deviation, 86 cells/HPF; and 95% confidence interval (CI), 66–141 cells/HPF. Assessment of the infiltration of CD8 cells showed a lower density than CD3 cells with a mean density of 87 cells/HPF. Median was 34 cells/HPF; range 2–317 cells/HPF; standard deviation 43 cells/HPF; and 95% CI, 44–129 cells/HPF.

### Statistics

**Figure 2.**
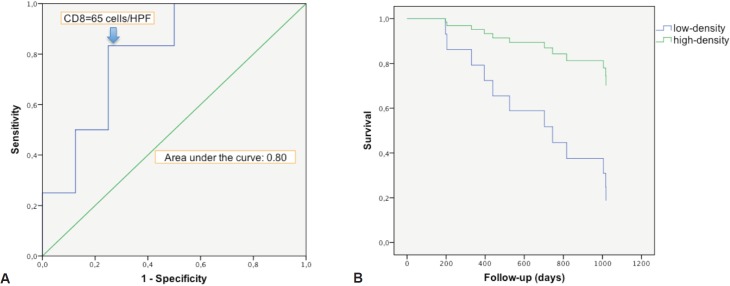
Survival analysis in relation with CD8 cells infiltration. a. ROC curve of survival predicted by CD8 density. The Euclidean shortest distance link the graph top left to the ROC curve point corresponding to a cell density of 65 cells/HPF. b. Kaplan–Meier cancer-specific survival curve. After splitting the population between high and low CD8 density, around the 65 cells/HPF value, high infiltration appeared to be protective for cancer-specific death (p = 0.034).

There was a strong correlation between the mean densities of CD3 and CD8 cells (*p* = 0.0001) with a linear relation between these two data sets (*p* = 0.0001). There was a linear relation between the variable survival time from first TURBT and mean density of CD3 cells (*p* = 0.02) and CD8 cells (*p* = 0.0238). When we compared survivor versus non-survivor groups, mean densities of CD3 cells (*p* = 0.048) and CD8 cells (*p* = 0.028) were significantly different. The CD3 and CD8 areas under the ROC curves were respectively of 0.78 and 0.80 (Figure 2a). The cell densities thresholds to separate groups had been set at 97 cells/HPF (CD3) and 65 cells/HPF (CD8). Survival analysis showed significantly better survival among CD3- and CD8-infiltrated tumours (*p* = 0.047 and *p* = 0.034, respectively) (Figure 2b). Using Cox univariate analysis (Table 2), CD3 cells (*p* = 0.034) and CD8 cells (*p* = 0.034) were significant predictors of survival, low TILS density being associated with a cancer-specific mortality risk. None of the other variables analysed were significant on univariate analysis, probably because of the small set of data (grade was not tested since all the tumours were high-grade, T stage on CT scan *p* = 0.81, N stage on CT scan *p* = 0.88, age *p* = 0.87, body mass index *p* = 0.96, gender *p* = 0.27, treatment (cystectomy versus other treatments) *p* = 0.35). Thus there was no reason to perform a multivariate analysis.

## Discussion

The results from this study suggest a significant relationship between the infiltration of Th1 reaction cells (CD3 and CD8) into the invasion margin of bladder tumour and a better survival in MIBC. It emphasises the importance of a cytotoxic immune reaction from ‘host’ against the tumour in bladder cancer prognosis (CD3 and CD8 lymphocytes mean density/HPF among survivor versus non-survivors respectively, of 158/HPF versus 69/HPF, and 146/HPF versus 45/HPF).

Many studies have reported a good prognostic value of high infiltration in Th1-related cells for various primary tumours such as melanoma, breast, prostate, lung, and colorectal cancers [10–16]. A 2012 review from Fridman *et al* [17] reported results from 124 articles dealing with cancer clinical outcomes and immune contexture. Among 60 articles focusing on TIL CD8 infiltration and 14 different cancer locations, 57 confirmed this association. Among 15 articles studying the association between other tumour-infiltrating cells linked with the Th1 reaction and prognosis of ten different primary tumour locations, 14 reported a good prognosis in highly infiltrated tumours.

To our knowledge, only one study has focused on the specific link between TIL CD8 and bladder cancer prognosis. In 2007, Sharma *et al* reported an analysis of 69 bladder tumour specimens from Memorial Sloan-Kettering Cancer Centre (New York, NY, USA) [8]. Thirty-three recurrences occurred in the cohort, and 15 patients died. A strong association between high central tumour TIL CD8 density and survival was shown for MIBC. Those results are concordant with ours. However, some differences should be noted between the two studies. Sharma *et al* focused on central-tumour infiltration, whereas we focused on peri-tumoural stromal infiltration. This is certainly linked to the original material investigated. Their group studied cystectomy material where the whole tumour specimen is available, whereas we decided to study TURBT material. We believe it is more relevant to base the analysis on this material because a potential immunoscore would have to predict outcomes early in the diagnosis, that is, at the first TURBT, and not at the treatment stage of a radical cystectomy. Furthermore, patients undergoing radical cystectomy have often received previous treatment before the procedure. More specifically, many of them probably received immunotherapy with BCG instillation into the bladder for NMIBC. These data are not clearly presented in the work by Sharma *et al*, although it was mentioned that 19 patients received neoadjuvant chemotherapy. At this point, we do not know how previous immunologic and/or chemotherapy treatment might have had an impact on TIL CD8 infiltration. For this reason, we decided to select only naïve patients for BCG and chemotherapies. The consequence of this inclusion criteria is that patients included in our cohort are less numerous yet more homogeneous than those analysed by Sharma *et al* TIL density in the present work was not associated with previous immunotherapies or chemotherapies.

Our study had various limitations including a retrospective design, probably causing selection biases. Although we had good results on univariate analysis, the power of our statistical analysis was limited by the small size of our cohort, and we could not establish a significant association between the already known criteria for the clinical outcomes of bladder cancer and survival. Finally, immunohistochemistry analysis is notoriously subject to interpretation bias. We tried to limit this bias by using a double-blind reading of the material. A solution would be an automated method to count TIL density into margin invasions, as reported by Galon in the development of an immunoscore to predict the clinical outcome of colorectal cancer [16].

Our results, associated with many other related studies, seem to argue for the universal prediction value of Th1 immune contexture in cancer clinical outcomes and could pave the way for larger validation studies. TNM stage, tumour grading, and most of the validated markers in oncology are used to lead treatment strategy from noninvasive to invasive surgical procedures, from topical to systemic treatments, and from simple to multimodal strategies, depending on the severity of the cancer classified by those markers. We believe that studying patient response to the tumour in addition to intrinsic tumour features may provide a better prognostication of bladder cancer patients.

- With regard to analysis of bladder muscle invasion based on TURBT material, we know that 4–25% of NMIBC diagnosed on first TURBT are under-staged and confirmed as MIBC when a second-look TURBT is performed [18,19].
- TNM stage is initially difficult to appreciate clinically since the tumour is usually not palpable [20], but neither with radiology. Indeed, local invasion imaging is not specific, at least for stages < T3b [21, 22]; and the specificity of lymph node enlargement on imaging is really poor under 8 mm [23, 24].
- Tumour grading is an important marker for predicting recurrence and progression in NMIBC. Unfortunately, it cannot apply to MIBC, and reproducibility is not good from one pathologist to another [7].

We believe that an immunoscore in bladder cancer could be a relevant marker and would have specific and practical applications. For NMIBC, improving pathologic techniques to discriminate high versus low TIL CD3/CD8 infiltration in pT1 stage tumours would be an invaluable tool in selecting appropriate therapeutic modalities. The determination of when to discontinue BCG and implement a more aggressive therapy is the hardest decision faced by urologists who manage high-risk NMIBC patients. Although this hypothesis could not be verified in this study, we can expect TIL density to be a logical marker to predict response to treatment before BCG instillation. For MIBC, the rising place of neoadjuvant chemotherapy prior to cystectomy is supported by various meta-analysis that confirmed the survival benefit of this treatment but with a limited improvement in absolute survival (5–10%) [25–27]. An immunoscore could be a relevant tool to discriminate a patient who would potentially most benefit from this treatment strategy.

Another important potential application of an immunoscore in bladder cancer would be to predict response to new immunotherapy. The place of new immune systemic treatments targeting cytotoxic response to sustain it, such as anti-cytotoxic T-lymphocyte associated protein 4 (CTLA4) and anti-programmed death receptor-1 (PD1) therapies, offers a new paradigm in oncology. Clinical trials have proven the efficacy of those treatments in melanoma [28], and many clinical trials are assessing those treatments in various cancers. One study demonstrated the feasibility of neoadjuvant treatment using ipilimumab (anti-CTLA4) prior to cystectomy for 12 patients harbouring bladder cancer [29]. The ability of TIL CD3/CD8 to predict response to these new treatments would be a clinically pertinent research endeavour.

## Conclusion

Our study emphasises the potential of an immunoscore in bladder cancer as a prognostic marker. Our results confirmed the feasibility of TIL CD3 and CD8 density measurements in the invasive margins of MIBC using immunohistochemistry techniques and their significant association with clinical outcomes. We believe further validation studies would be worth performing.

## Figures and Tables

**Table 1.** Clinical data manuscript Th1.

PATIENT	PATHOLOGY	GENDER	AGE	SMOKING	CIRCUMSTANCES	T STAGE (CT-SCAN)	N STAGE (CT-SCAN)	PROGRESSION	RECURRENCE	CYSTECTOMY	RADIATION	CHEMOTHERAPY	MORTALITY	FOLLOWUP (days)	T3 (mean)	T8 (mean)
1	MIBC	1	59.8	1	HAEMA-TURIA	2	0	0	0	1	0	0	0	578	99	75
2	MIBC	1	73.9	1	HAEMA-TURIA	3	0	1	1	1	0	1	1	1005	129	157
3	MIBC	2	83.7	np	HAEMA-TURIA	2	0	0	1	0	0	0	0	1705	317	196
4	MIBC	1	76.8	1	HAEMA-TURIA	3	0	0	0	1	0	1	0	1879	233	243
5	MIBC	2	57.8	0	HAEMA-TURIA	2	0	1	1	1	0	1	1	745	95	13
6	MIBC	2	70.4	1	HAEMA-TURIA	3	0	0	0	1	0	1	0	1033	229	138
7	MIBC	1	80.2	0	HAEMA-TURIA	2	0	1	1	0	1	1	1	1017	58	11
8	MIBC	1	63.5	np	FIBROS-COPY	2	0	1	1	1	0	0	1	197	49	37
9	MIBC	1	60.9	1	HAEMA-TURIA	2	0	1	1	1	0	1	0	1102	20	8
10	MIBC	1	88.1	np	ULTRA-SOUND	2	0	1	1	0	0	1	1	703	10	3
11	MIBC	1	74.9	1	ULTRA-SOUND	3	0	1	1	1	0	1	1	817	42	30
12	MIBC	1	65.8	np	HAEMA-TURIA	2	2	1	1	1	0	1	1	439	85	25
13	MIBC	1	75.5	1	ULTRA-SOUND	2	0	1	1	0	1	1	1	204	26	14
14	MIBC	1	65.2	1	ULTRA-SOUND	2	0	0	0	1	0	1	0	1631	200	183
15	MIBC	1	79.9	1	CT-SCAN	3	0	1	1	0	1	1	1	525	184	181
16	MIBC	1	65.2	0	ULTRA-SOUND	2	1	0	0	1	0	0	0	1316	108	310
17	MIBC	1	65.7	np	HAEMA-TURIA	2	1	1	1	1	0	1	1	395	45	7
18	MIBC	1	61.7	1	CT-SCAN	3	0	1	1	1	0	1	1	330	82	55
19	MIBC	1	69.3	1	HAEMA-TURIA	3	0	0	0	1	0	1	1	1018	29	7
20	MIBC	1	67.9	1	CT-SCAN	2	0	0	0	1	0	0	0	1856	56	19
21	NMIBC	1	63.5	np	FIBROS-COPY	np	np	1	1	1	0	0	1	197	49	37
22	NMIBC	1	23.5	1	HAEMA-TURIA	np	np	1	1	1	0	1	1	753	90	101
23	NMIBC	1	74.1	np	ULTRA-SOUND	np	np	0	1	0	0	0	0	1931	108	113
24	NMIBC	1	60.7	0	HAEMA-TURIA	np	np	1	1	1	0	1	0	1780	50	107
25	NMIBC	2	83.7	0	HAEMA-TURIA	np	np	0	0	0	0	0	0	1337	92	139
26	NMIBC	1	69.4	1	HAEMA-TURIA	np	np	0	0	0	0	0	0	317	145	53
27	NMIBC	1	65.2	1	CT-SCAN	np	np	0	0	0	0	0	0	1535	105	39
28	NMIBC	1	69.6	1	HAEMA-TURIA	np	np	0	0	0	0	0	0	1765	64	49
29	NMIBC	1	86.4	np	CT-SCAN	np	np	0	0	0	0	0	0	1324	181	161
30	NMIBC	1	65.6	1	HAEMA-TURIA	np	np	0	0	0	0	0	0	1513	138	39

**Table 2.** Cox analysis results.

Factor analysed	*p*
CD3 cell infiltration (continuous)	0.026
CD3 cell infiltration (nominal, threshold 97 cells/HPF)	0.034
CD8 cell infiltration (continuous)	0.020
CD8 cell infiltration (nominal, threshold 65 cells/HPF)	0.034
T stage (CT-scan evaluation)	0.813
N stage (CT-scan evaluation)	0.880
Treatment (cystectomy versus other)	0.350
Age (continuous)	0.874
Age (nominal, threshold 69 years)	0.865
Body Mass Index	0.982
Gender	0.271
